# Monitoring changes in human activity during the COVID-19 shutdown in Las Vegas using infrasound microbarometersa)

**DOI:** 10.1121/10.0003777

**Published:** 2021-03-15

**Authors:** Elijah J. Bird, Daniel C. Bowman, Douglas R. Seastrand, Melissa A. Wright, Jonathan M. Lees, Fransiska K. Dannemann Dugick

**Affiliations:** 1 Geophysical Detection Programs, Sandia National Laboratories, 1515 Eubank Southeast, MS 0404, Albuquerque, New Mexico 87123, USA; 2 Advanced Technologies Division, Nevada National Security Site, P.O. Box 98521, Las Vegas, Nevada 89193-8521, USA; 3 Nevada National Security Site, P.O. Box 98521, Las Vegas, Nevada 89193-8521, USA; 4 Department of Geological Sciences, 104 South Road, CB #3315, University of North Carolina, Chapel Hill, North Carolina 27599-3315, USA

## Abstract

While studies of urban acoustics are typically restricted to the audio range, anthropogenic activity also generates infrasound (<20 Hz, roughly at the lower end of the range of human hearing). Shutdowns related to the COVID-19 pandemic unintentionally created ideal conditions for the study of urban infrasound and low frequency audio (20–500 Hz), as closures reduced human-generated ambient noise, while natural signals remained relatively unaffected. An array of infrasound sensors deployed in Las Vegas, NV, provides data for a case study in monitoring human activity during the pandemic through urban acoustics. The array records a sharp decline in acoustic power following the temporary shutdown of businesses deemed nonessential by the state of Nevada. This decline varies spatially across the array, with stations close to McCarran International Airport generally recording the greatest declines in acoustic power. Further, declines in acoustic power fluctuate with the time of day. As only signals associated with anthropogenic activity are expected to decline, this gives a rough indication of periodicities in urban acoustics throughout Las Vegas. The results of this study reflect the city's response to the pandemic and suggest spatiotemporal trends in acoustics outside of shutdowns.

## I. INTRODUCTION

Natural phenomena and human activities can generate low frequency sound waves that may propagate vast distances. Within geophysics communities, such signals below 20 Hz are known as infrasound (Le Pichon *et al.*, 2010). Past investigations of anthropogenic acoustic signals in the infrasound range have focused on discrete sources such as chemical and nuclear explosions (Park *et al.*, 2014; Park *et al.*, 2018), rockets (Evers *et al.*, 2018), aircraft (Evers, 2005; Le Pichon *et al.*, 2002), bridges (Donn *et al.*, 1974; Whitlow *et al.*, 2019), and wind turbines (Marcillo *et al.*, 2015). In contrast, little attention has been paid to the characteristics of ambient urban acoustics in the low frequency audio to infrasound range. Although there has been some work on sources and propagation characteristics (e.g., McComas *et al.*, 2018; Albert and Decato, 2017), urban infrasound is more often seen as an annoyance than as an asset. The COVID-19 pandemic and associated shutdown shows how urban infrasound and low frequency audio signals can track changes in human activity. This paper is concerned with monitoring patterns of human activity via the acoustic signals they generate [as in McComas *et al.* (2018)] rather than changes in the human perception of the sonic environment (e.g., Brown *et al.*, 2016).

**FIG. 1.**
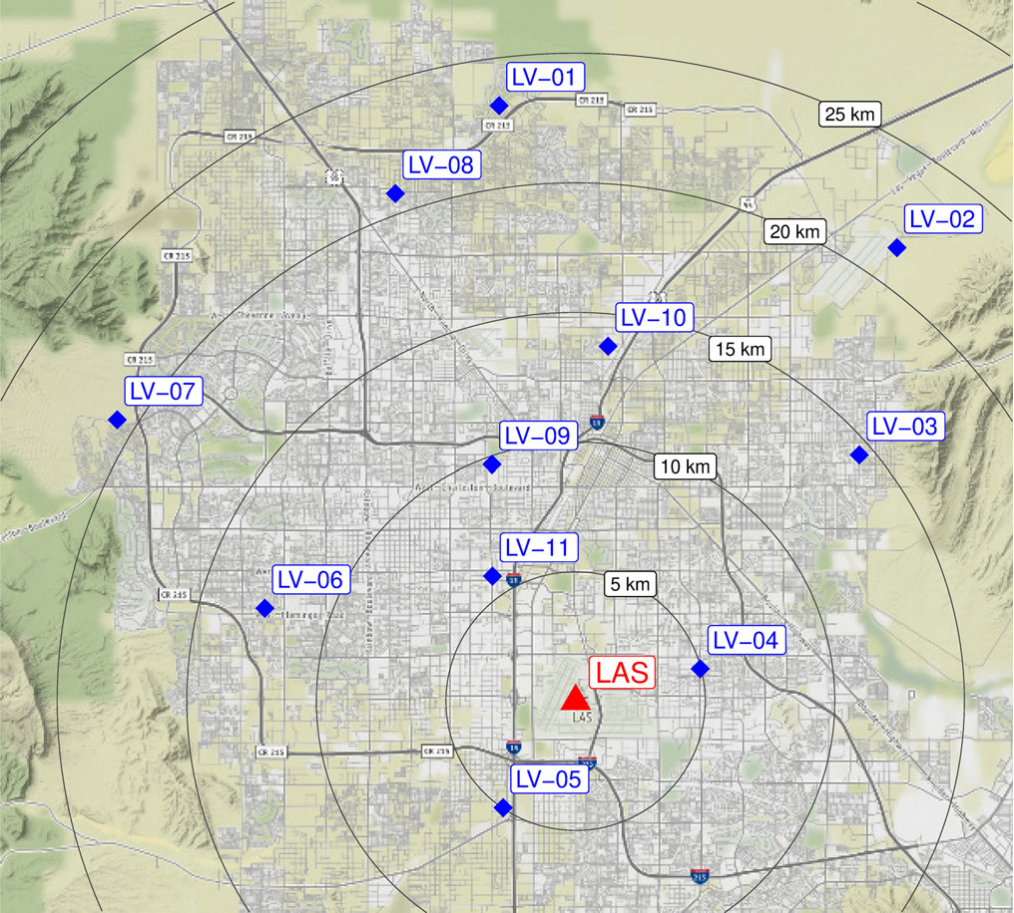
(Color online) Map of LVIA stations. Range circles note distance from LAS.

**FIG. 2.**
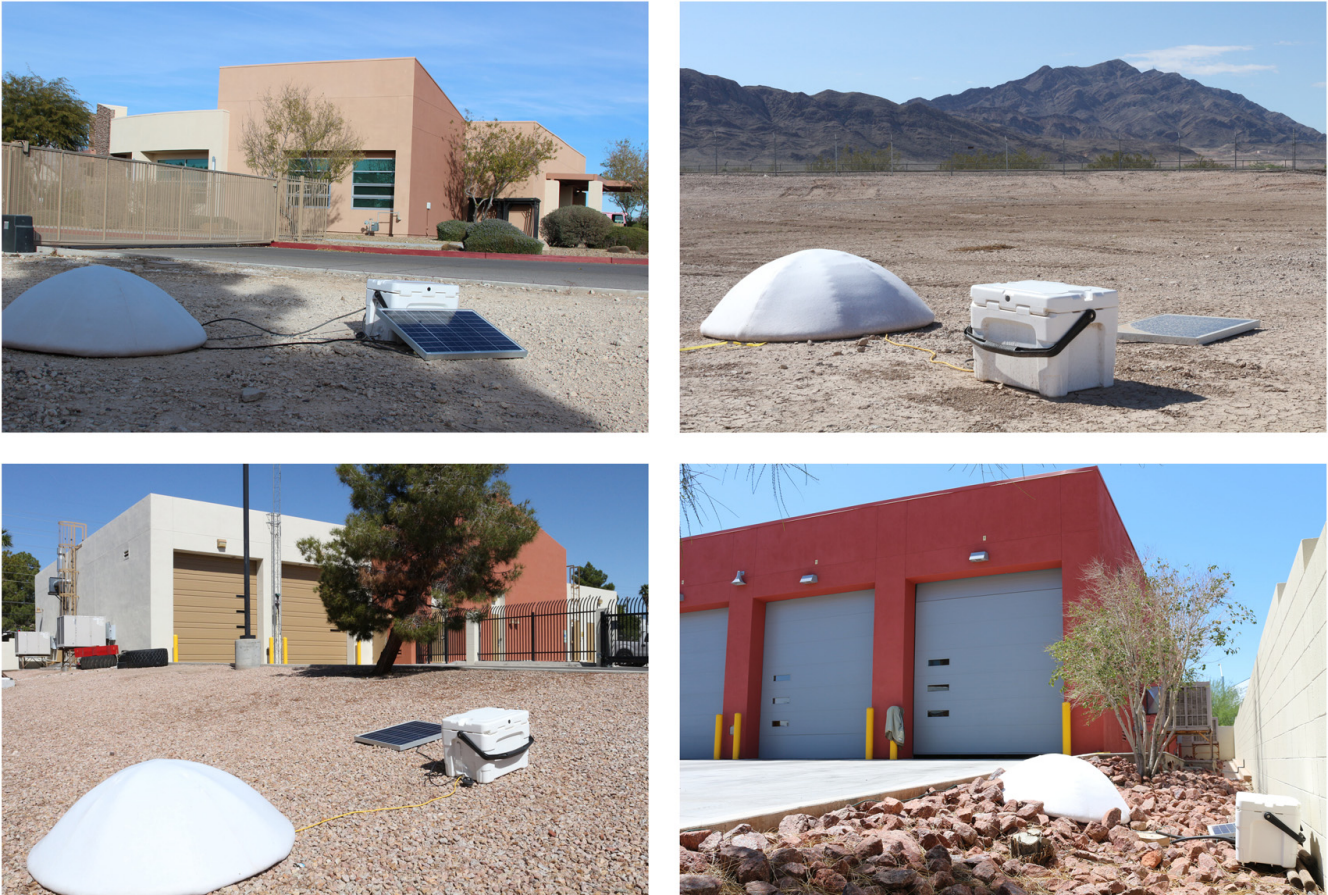
(Color online) Various stations on the array: LV01 (top left), LV02 (top right,) LV04 (bottom left), and LV11 (bottom right).

**FIG. 3.**
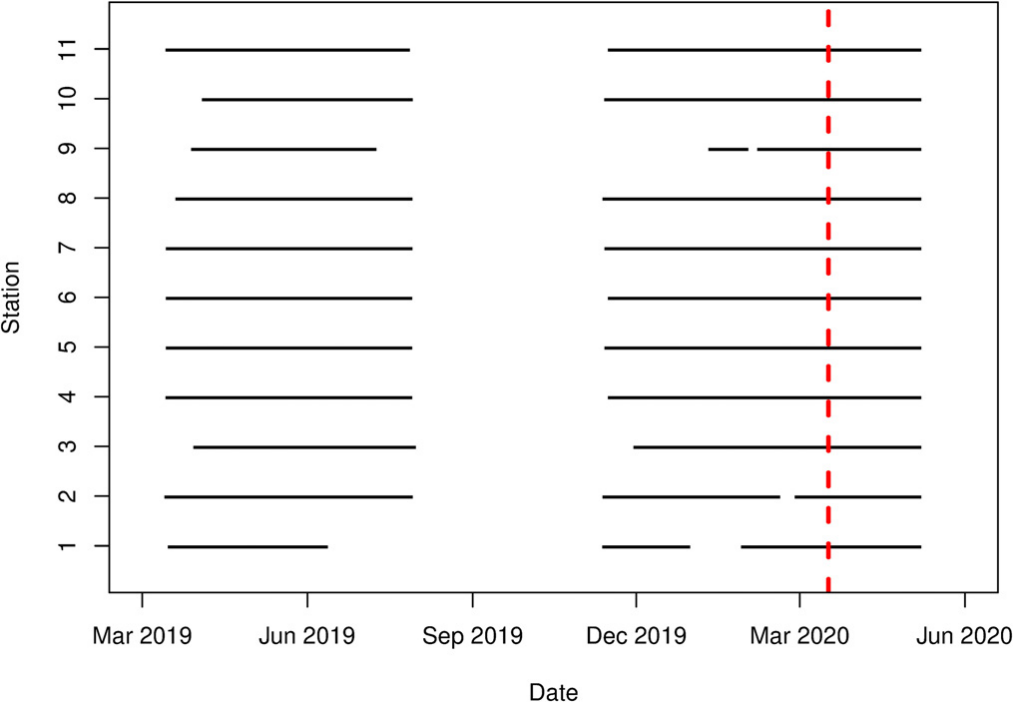
(Color online) Temporal coverage of each station on the LVIA. The dotted line indicates the date when restrictions were placed on nonessential businesses.

The Las Vegas Infrasound Array (LVIA) project was conceived to characterize the ambient low frequency audio and infrasound environment of a major urban area and to determine whether variations in its characteristics could be linked to changes in atmospheric state. To this end, we deployed 11 infrasound stations throughout the Las Vegas metro area (Fig. 1). Each station consisted of a single seismically decoupled digital Hyperion (Tupelo, MS) microbarometer sampling at 1000 Hz (although one station recorded at 400 Hz for the first few months) (Fig. 2). The network was brought online in March 2019 and remains in place as of this writing, with some time gaps when the sensors were needed for other projects (Fig. 3). Midway through this deployment, the COVID-19 pandemic caused a dramatic shift in human activity across the globe (Andersen *et al.*, 2020). This immense, unplanned “experiment” has highlighted the many ways in which the anthropocene has impacted the planet. For example, cities in China saw reductions in NO_X_ emissions by 20%–50% during shutdown periods (Ding *et al.*, 2020). Similarly, CO_2_ emissions in the San Francisco Bay Area decreased by roughly 30% following shelter-in-place orders (Turner *et al.*, 2020). Ambient seismic noise in the 4–14 Hz range lessened globally, with the most intense effects in populous areas (Lecocq *et al.*, 2020). Acoustic power on A-equivalent instruments, which mimic the perception of sound by humans, was shown to have similar declines in Lima, Peru (Montano and Gushiken, 2020). However, changes in urban acoustics were not uniform: Traffic noise patterns showed no clear trend during the shutdown in Buenos Aires (Said *et al.*, 2020), and church bell activity increased in some areas and decreased in others in Australia (Parker and Spennemann, 2020).

Las Vegas had its first reported case of the virus on March 5, 2020 (Star Advertiser Associated Press, 2020). On March 12, 2020, a state of emergency was declared for the state of Nevada (Sadler, 2020). March 17 saw the temporary closure of nonessential businesses (Messerly *et al.*, 2020). Nevada Governor Sisolak outlined the term “nonessential businesses” as including casinos, gyms, and dine-in restaurants, among other establishments (Snyder *et al.*, 2020). This order was in effect until May 9, when some nonessential businesses, including restaurants and retailers, reopened at reduced capacity (McGinness, 2020). The city's casinos remained closed until June 4, when some reopened (Bradford, 2020). As of December 17, this information is accessible at the listed sources.

The presence of the LVIA before and during the COVID-19 pandemic provides a unique opportunity to examine human patterns of life through an infrasound lens. It has allowed us to determine which frequencies are most impacted by human activity and to evaluate spatial and temporal trends in anthropogenic noise across the Las Vegas region. We discuss these patterns in terms of proximity to key regions in the city (e.g., the airport, the casinos on the Las Vegas Strip, residential areas, construction sites, etc.). These results contribute to a greater understanding of the urban infrasound and low frequency audio environment, in the context of the human activities that contribute to it. Our results have implications for focused observations of specific activities of interest within a noisy cityscape, such as acoustic monitoring of nuclear reactors and other vital infrastructure (Marcillo *et al.*, 2020; McComas *et al.*, 2020) or traffic patterns (Albert and Decato, 2017).

## II. METHODS

The high sampling rates of the LVIA result in a massive raw data set for which analysis is prohibitively time intensive. Data are thus condensed to a more tractable structure, consisting of an average power value within 18 frequency bands of interest for each hour and station (Table I). The choice of logarithmically scaling frequency bands is consistent with past work in the infrasound range (Matoza *et al.*, 2013). The following process is applied to each hour of a given station's associated time series.

**TABLE I.** Minimum, median, and maximum frequency values within each frequency band.

Band #	*f_min_* (Hz)	*f_center_* (Hz)	*f_max_* (Hz)	Band #	*f_min_* (Hz)	*f_center_* (Hz)	*f_max_* (Hz)
1	0.000	0.002	0.004	10	1.000	1.500	2.000
2	0.004	0.006	0.008	11	2.000	3.000	4.000
3	0.008	0.012	0.016	12	4.000	6.000	8.000
4	0.016	0.023	0.031	13	8.000	12.000	16.000
5	0.031	0.047	0.062	14	16.000	24.000	32.000
6	0.062	0.094	0.125	15	32.000	48.000	64.000
7	0.125	0.187	0.250	16	64.000	96.000	128.000
8	0.250	0.375	0.500	17	128.000	192.000	256.000
9	0.500	0.750	1.000	18	256.000	378.000	500.000

Using the discrete Fourier Transform (DFT), we calculate the single-sided power spectrum in Pa^2^/Hz for the time series (Davis, 2002). A conversion to dB (using a reference amplitude of 4 × $10^{-10}$ Pa^2^/Hz) aids in reducing the effects of outliers on the distributions. Within each frequency band, we determine the mean of the dB power spectrum. This process compacts the time series for each hour and station, which typically contain 3 600 000 observations, to 18 relevant statistics. In doing so, we retain no information of the sound character within frequency bands or of how power in said bands varies within the hour. However, such information is not crucial to our broad, long-term analysis of ambient acoustics.

## III. ANALYSIS

### A. Estimating overall drop in power

Estimates in the decline of acoustic power during the shutdowns require baseline distributions of power levels at each station and frequency. The first 29 weeks for which data were recorded (all available weeks of data prior to February 2020) act as this baseline. Monday at 12:00 a.m. PDT/PST is used as the start of the week.

The mean power for each station (*i*) and frequency band (*j*) during the *k*th hour of the *l*th week of data (determined above) is ${\bar{X}}_{\mathrm{ijkl}}$. Noting that there are 168 h in each week, we calculate the mean power for each station, frequency band, and week of data as

$${\bar{X}}_{\mathrm{ijl}}=\frac{1}{168}\sum_{k=1}^{168}{\bar{X}}_{\mathrm{ijkl}}.$$ (1)

Similarly, the mean power for each station and frequency band, during the baseline period is

$${\bar{X}}_{ij_{\text{baseline}}}=\frac{1}{29}\sum_{l=1}^{29}{\bar{X}}_{\mathrm{ijl}}.$$ (2)

Finally, the standard deviation (SD) of power from week to week, for each band and station is

$$s_{ij_{\text{baseline}}}=\sqrt{\frac{\sum_{l=1}^{29}{\left({\bar{X}}_{\mathrm{ijl}}-{\bar{X}}_{ij_{\text{baseline}}}\right)}^{2}}{28}}.$$ (3)

Distributions of ${\bar{X}}_{\mathrm{ijl}}$ values are expected to approach normality, by the central limit theorem (Davis, 2002). As such, ${\bar{X}}_{ij_{\text{baseline}}}$ and $s_{ij_{\text{baseline}}}$ provide an estimate on the distribution of mean weekly power values for frequency band *j* and station *i*. We determine where later weeks (i≥30), particularly those surrounding shutdowns, fall in the baseline distribution:

$$\alpha_{\mathrm{ijl}}=\frac{{\bar{X}}_{\mathrm{ijl}}-{\bar{X}}_{ij_{\text{baseline}}}}{s_{ij_{\text{baseline}}}},$$ (4)

where *α_ijl_* is the distance in baseline SDs between ${\bar{X}}_{\mathrm{ijl}}$ and the baseline mean (for station *i* and band *j*). As a consequence of approximate normality, *α_ijl_* gives a rough estimate on where a given value of ${\bar{X}}_{\mathrm{ijl}}$ would fall into the baseline distribution. If the distribution of power levels remains constant following shutdowns, only 2.3% of *α* values should be less than –2, and only 6.7% of *α* values should be less than –1.5 (Davis, 2002). Similarly, an analog to *α_ijl_* values that considers average power across the 11 stations can be computed as follows:

$${\bar{X}}_{jl}=\frac{1}{11}\sum_{i=1}^{11}\frac{1}{168}\sum_{k=1}^{168}{\bar{X}}_{\mathrm{ijkl}},$$ (5)

$${\bar{X}}_{j_{\text{baseline}}}=\frac{1}{29}\sum_{l=1}^{29}{\bar{X}}_{jl},$$ (6)

$$s_{j_{\text{baseline}}}=\sqrt{\frac{\sum_{l=1}^{29}{\left({\bar{X}}_{jl}-{\bar{X}}_{j_{\text{baseline}}}\right)}^{2}}{28}},$$ (7)

$$\alpha_{jl}=\frac{{\bar{X}}_{jl}-{\bar{X}}_{j_{\text{baseline}}}}{s_{j_{\text{baseline}}}}.$$ (8)

**FIG. 4.**
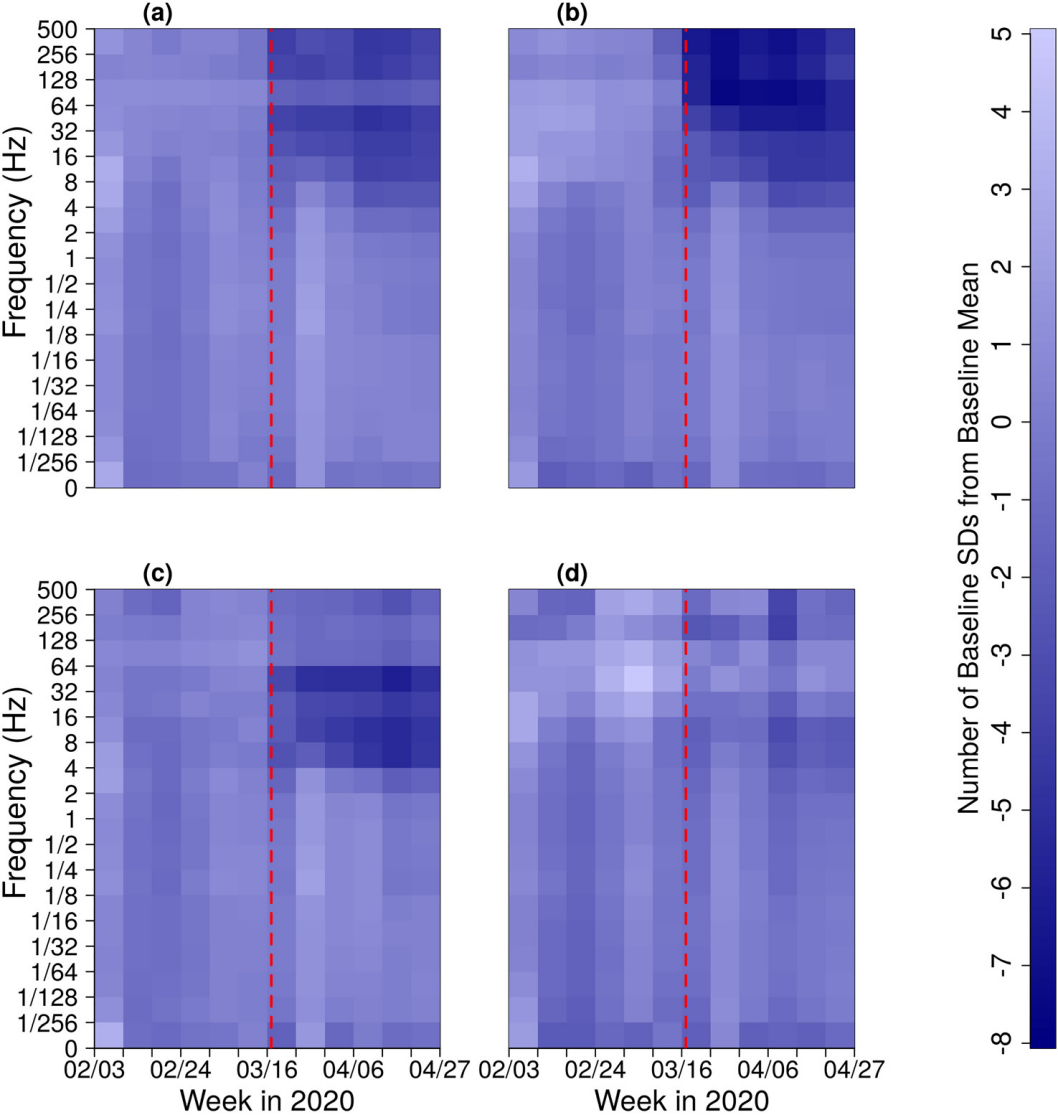
(Color online) *α* values (i.e., number of baseline SDs below baseline mean power) (a) for mean power levels across all stations; (b) at LV05, a station close (within 5.0 km) to LAS; (c) at LV10 (13.8 km from LAS); (d) at LV07, a station distal (20.8 km) from LAS. *α* values in the 8–500 Hz range at stations close to the airport dropped considerably after March 16, 2020. Darker blues distinguish a decline in power. The dashed, vertical lines indicate March 17, the date on which nonessential businesses were ordered to close.

Within the 8–500 Hz range, *α_jl_* values are consistently less than –1.5 following March 16, 2020. In the same range of frequencies, some stations more central to Las Vegas (e.g., LV05) see *α_ijl_* values consistently below –2 during shutdowns (Fig. 4). Notably, the lower end of the 8–500 Hz range lies at infrasonic frequencies. Certain stations see drops in acoustic power at even lower frequencies. After March 16, *α_ijl_* values in the 4–8 Hz range at LV10 remain below –1.5. Once again considering averages across the array, the most intense relative drop in acoustic power occurs in the 32–64 Hz range, where the mean of *α_jl_* during shutdowns is –4.2. Past work on anthropogenic infrasound has found that peak noise levels in non-industrial urban areas occur in the 32–80 Hz range, matching closely to the band in which we observe the greatest declines in power (Albert and Decato, 2017). The frequency band with the greatest decline in power varies between the stations but consistently sits in the 8–256 Hz range.

**FIG. 5.**
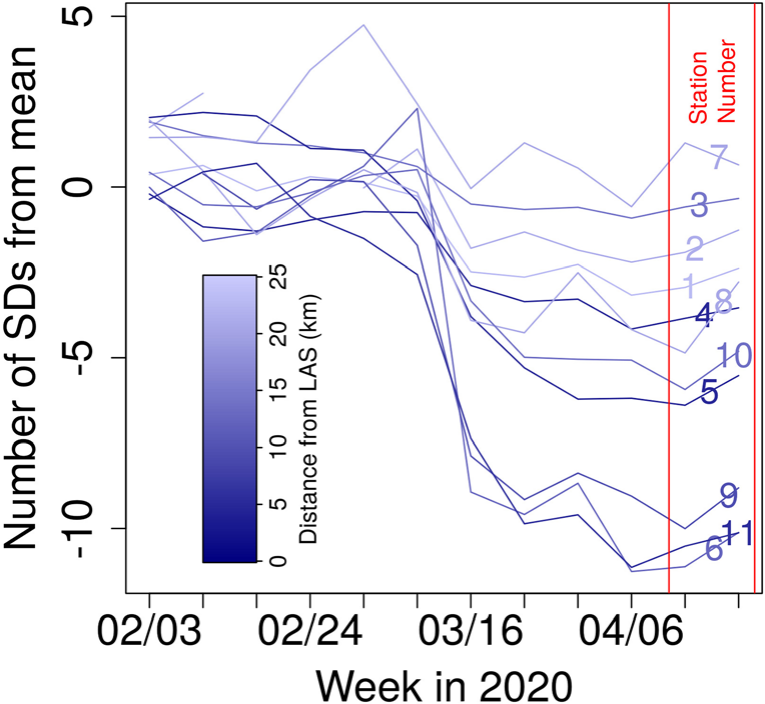
(Color online) $\alpha_{i15l}$ values (i.e., number of baseline SDs from baseline mean in the 32–64 Hz range) at LV array stations over time. Station numbers are listed at the right side of the plot. Lines are color coded by distance from LAS. Declines in acoustic power were especially pronounced near the center of the city. Station number is indicated between the solid, vertical lines.

We expect the impact of COVID-related shutdowns on acoustic power to vary spatially. We particularly expect to see differences between urban (e.g., stations LV04, LV05, and LV11) and less centrally located stations (e.g., stations LV01, LV02, and LV07), as noise is expected to increase with heightened human activity. In quantifying the degree to which each station is centrally located, we treat McCarran International Airport (LAS) as the center of acoustic activity in Las Vegas. This is reasonable, as the airport is proximal to the Vegas Strip, so the choice takes sound from hotels and casinos into account, in addition to the airport itself. It is worth noting that the airport is not geographically central, with much of the city and array lying to its north. Following shutdowns, stations closer to the center of the city see greater declines in power (Fig. 5). During the week of the shutdown period, the mean $\alpha_{i15l}$ value for the five closest stations is −7.5, whereas the six most distal stations observe a mean $\alpha_{i15l}$ value of –2.2 (where $\alpha_{i15l}$ indicates *α_ijl_* values for the 32–64 Hz band).

### B. Daily trends in acoustic power

Anthropogenic infrasound tends to fluctuate with a daily period, reflecting typical trends in human activity (Albert and Decato, 2017). However, naturally generated infrasound also takes on a diurnal trend, due in part to increased wind speeds during daylight hours (Bowman *et al.*, 2005). As such, broad trends in natural and anthropogenic acoustic power can be difficult to separate. In this case, we expect the COVID-19 shutdown to distinguish sources, as natural signals should persist throughout the shutdown while anthropogenic signals should decline, similarly to trends observed in ambient seismic data (Lecocq *et al.*, 2020). To gauge the scale of this reduction, we compare hourly changes in acoustic power during the shutdowns (in the most affected frequency bands) to a pre-shutdown baseline. This allows us to examine when nonessential human activity typically takes place, as the only signals to decline should be those associated with nonessential activity (“nonessential activity” refers to activity the Las Vegas population does not take part in during shutdowns, either due to personal choice or government-mandated restrictions).

First, estimates of daily power fluctuations during shutdowns are investigated. Below 1 Hz, natural signals are typically more pronounced than anthropogenic signals (Albert and Decato, 2017). Further, in the LVIA dataset, power at frequencies below 1 Hz tends to exceed that at frequencies above 1 Hz. Thus, to prevent natural signals from dominating our results, we isolate the higher frequency bands that are noticeably affected by COVID-related shutdowns. Based on the analysis above, declines in power are most prominent in the 8–500 Hz range and are present throughout the period from March 30, 2020 to April 26, 2020. These dates were chosen as they fall within the shutdown of nonessential businesses (McGinness, 2020; Messerly *et al.*, 2020). During this timespan and for frequency bands in the 8–500 Hz range, the weekly mean power across the array remains at least 1.5 SDs below the baseline weekly mean power. This is not the case for frequency bands below 8 Hz.

Then, considering ${\bar{X}}_{\mathrm{imn}}$, the mean power in the 8–500 Hz frequency band at the *i*th station and during the *m*th hour of the *n*th day after March 30, 2020, the mean across the 28 days in question is

$${\bar{X}}_{im_{2020}}=\frac{1}{28}\sum_{n=0}^{27}{\bar{X}}_{imn_{2020}}.$$ (9)

Similarly, the mean power during the *m*th hour of the day, averaged over the stations, is

$${\bar{X}}_{m_{2020}}=\frac{1}{10}\sum_{\begin{array}{c} n=1\\ n\neq 3 \end{array}}^{11}{\bar{X}}_{im_{2020}}.$$ (10)

Repeating this process for 2019, with *n* now describing the number of days after April 1, 2019 (for 2019, we start on April 1, rather than March 30, as this allows the reference period to begin on a Monday at 12:00 a.m. PDT, as is the case for the 2020 period), we obtain

$${\bar{X}}_{im_{2019}}=\frac{1}{28}\sum_{n=0}^{27}{\bar{X}}_{imn_{2019}},$$ (11)

$${\bar{X}}_{m_{2019}}=\frac{1}{10}\sum_{\begin{array}{c} n=1\\ n\neq 3 \end{array}}^{11}{\bar{X}}_{im_{2019}}.$$ (12)

LV03 is excluded in this analysis, as it recorded at 400 Hz throughout April 2019, preventing any insights into the 200–500 Hz range [hence, n≠3 in the discrete sums seen in Eqs. (10) and (12)].

**FIG. 6.**
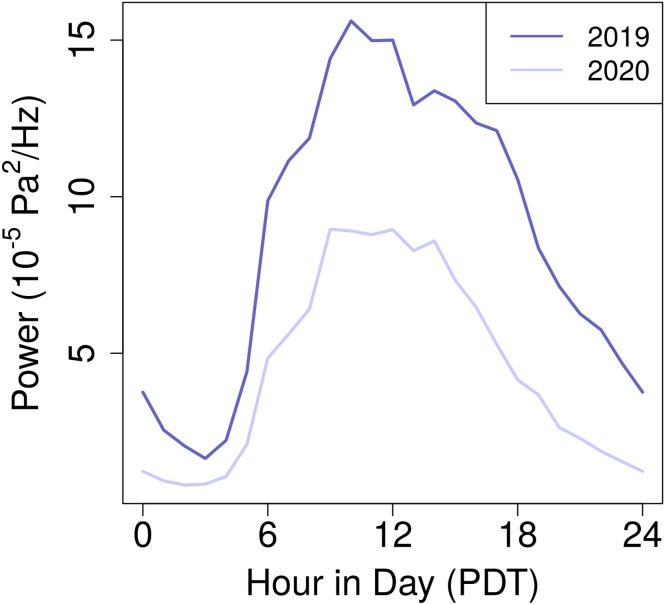
(Color online) April 2020 hourly acoustic power (${\bar{X}}_{m_{2020}}$) versus April 2019 hourly acoustic power (${\bar{X}}_{m_{2019}}$).

Figure 6 demonstrates that while mean hourly power is consistently lower in April 2020 relative to April 2019, acoustic power maintains similar daily trends in both timespans. Power peaks in the late morning and early afternoon (9 a.m.–3 p.m. PDT) and is lowest in the early morning (1–4 a.m. PDT). Albert and Decato (2017) observed diurnal changes in rural soundscapes, but their urban results were limited to “short time periods since permanent, secure sites were not available.” As such, this study acts as a first look into diurnal variations in urban acoustic power.

**FIG. 7.**
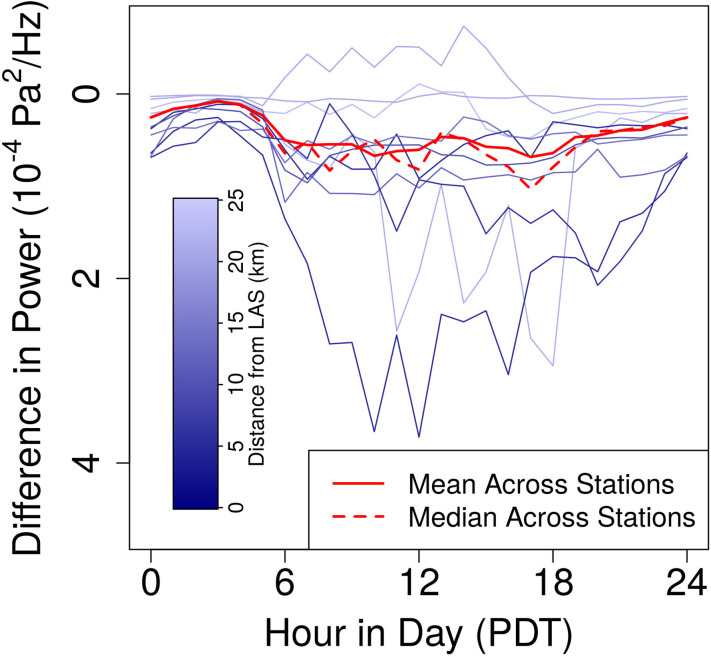
(Color online) Declines in power across all stations (${\bar{X}}_{m_{2019}}-{\bar{X}}_{m_{2020}}$) and for station *i* (${\bar{X}}_{im_{2019}}-{\bar{X}}_{im_{2020}}$), where i∈{1,2,4,5,…,11}, are plotted against time.

We assume that all natural signals stay roughly constant. This is reasonable given the long timespan that we average over. Further, past studies of ambient natural signals indicate that they vary most intensely with the time of day, season, and station (Bowman *et al.*, 2005), all of which remain constant in the comparison of 2019 and 2020 data. Then daily fluctuations in sound associated with nonessential anthropogenic activity can be approximated by subtraction of the 2020 results from those for 2019. These periodicities differ greatly between stations. Stations close to LAS consistently decline in power, but the times of greatest decline vary between stations from 11 a.m. to 11 p.m. Relative to these declines, power at stations farther from the airport typically drops very little during shutdowns, sometimes even increasing (Fig. 7).

## IV. CONCLUDING REMARKS

The results above give insights into the acoustics of Las Vegas, both during shutdowns and outside of the COVID-19 pandemic. Acoustic power dropped most intensely starting in the week of March 16, 2020, likely in response to the closures of nonessential businesses on March 17. Stations located close to LAS saw particularly dramatic effects. This is a predictable outcome, based on their proximity to both the airport and the city's casinos. Stations situated farther from LAS experienced relatively modest declines in acoustic power. Station LV07 saw higher than average acoustic power in the 32–128 Hz range. This is likely related to nearby construction during shutdowns, increasing acoustic power relative to the baseline period. The increase in power in this range is in line with past work on acoustics generated by construction, which demonstrated highest noise levels in the 50–160 Hz band. (Albert and Decato, 2017).

As stated earlier, the timing of drops in acoustic power between April 2019 and April 2020 is expected to roughly reflect the times at which nonessential activities take place. Daily trends in acoustic power vary greatly throughout the city. Even among stations closest to LAS, LV05 observes the greatest declines between 10 a.m. and 1 p.m., while LV11 sees peak declines from 9 to 10 p.m. This can be explained by the wide range of establishments surrounding the airport. LAS itself and more traditional businesses are likely to peak in activity in the middle of the day. The many nearby casinos, however, would be expected to experience the greatest activity at nighttime. Farther from the airport, most stations observe a less intense decrease in power, making trends difficult to discern. LV07, however, records an increase in power between 6 a.m. and 5 p.m., likely due to the aforementioned construction. The mean decline in power across the array was at its highest between 6 a.m. and 8 p.m., dipping slightly around 1–3 p.m. In averaging, the diversity of trends between stations is ignored, and a more general periodicity in human life across Las Vegas is determined.

Distance from LAS is not the definitive factor in determining the level of activity throughout Las Vegas. LAS is positioned in the southern portion of the city, so stations further north, such as LV08, may remain well within the bounds of the city but at a great distance from LAS. LV08 sees intense drops in power in the 16–64 Hz range. Another interesting case is LV02. While the station lies at the very edge of the city and is among the farthest from LAS, it is on Nellis Air Force Base, which is expected to produce high acoustic power. During shutdowns, LV02 experiences an intense drop in acoustic power between the hours of 11 a.m. and 7 p.m. The decline is greater than that at many stations close to LAS. This is expected to be largely associated with activity at the base.

This work indicates the value of further inquiries into low frequency, urban acoustics both during and outside of shutdowns. It would be worthwhile to investigate how acoustic power shifts as various safety measures are relaxed, such as those loosened on May 9 and June 4, 2020. Some nonessential businesses reopened during the former, but all casinos remained closed until the latter. As such, analysis could demonstrate the relative effect of each type of business on the city's acoustics, both spatially and temporally. This is outside the scope of our study, as the pandemic is still affecting human behavior, and an analysis of the LVIA's return to typical acoustic power would be premature. It may also be worthwhile to compare the acoustic record to metrics measuring human activity, such as traffic or flight data. Our analysis illuminates geographic and temporal trends in low frequency acoustics throughout Las Vegas, NV, and additional work with the LVIA dataset has the potential to clarify said trends.
